# Impact of exercise on cancer: mechanistic perspectives and new insights

**DOI:** 10.3389/fimmu.2024.1474770

**Published:** 2024-09-13

**Authors:** Ye Feng, Xingting Feng, Renwen Wan, Zhiwen Luo, Lijun Qu, Qing Wang

**Affiliations:** ^1^ School of Stomatology, Xuzhou Medical University, Xuzhou, Jiangsu, China; ^2^ Department of Sports Medicine, Huashan Hospital, Fudan University, Shanghai, China; ^3^ Department of Orthopaedics, Kunshan Hospital of Chinese Medicine, Kunshan, Jiangsu, China

**Keywords:** exercise, tumor microenvironment, cytokines, anti-cancer immunity, prevention, treatment

## Abstract

This review critically evaluates the substantial role of exercise in enhancing cancer prevention, treatment, and patient quality of life. It conclusively demonstrates that regular physical activity not only reduces cancer risk but also significantly mitigates side effects of cancer therapies. The key findings include notable improvements in fatigue management, reduction of cachexia symptoms, and enhancement of cognitive functions. Importantly, the review elucidates the profound impact of exercise on tumor behavior, modulation of immune responses, and optimization of metabolic pathways, advocating for the integration of exercise into standard oncological care protocols. This refined abstract encourages further exploration and application of exercise as a pivotal element of cancer management.

## 1 Introduction

The global burden of cancer continues to escalate, with millions of new cases diagnosed annually, which highlights the urgent need for effective prevention and treatment strategies. Recent statistics from major health organizations underscore a concerning rise in cancer incidence and mortality rates worldwide, compelling the medical community to explore innovative therapeutic modalities beyond traditional medical interventions (1, 2). Exercise oncology has emerged as a pivotal field of research, offering promising avenues for enhancing cancer prevention, treatment efficacy, and patient quality of life. The integration of exercise into oncological care is driven by a growing body of evidence that demonstrates the multiple benefits of physical activity for cancer patients. These benefits range from reducing the risk of cancer development and recurrence to alleviating the side effects of conventional cancer treatments such as chemotherapy and radiotherapy (3, 4). This review delves into the multifaceted advantages of exercise in the realm of cancer prevention and treatment. Consistent physical activity is demonstrated to not only mitigate the incidence and recurrence of cancer but also augment the efficacy of various cancer therapies, including surgery, radiotherapy, chemotherapy, and immunotherapy. Moreover, exercise significantly alleviates the adverse effects associated with cancer treatments such as fatigue, cancer cachexia, and cognitive impairments. Building upon these therapeutic supports, the subsequent sections delve into the broader implications of exercise on the tumor microenvironment, showcasing its profound impact on tumor angiogenesis, cytokine modulation, and overall tumor behavior. Here, ‘cancer cachexia’ refers to a complex syndrome involving muscle and weight loss, while ‘cognitive impairments’ relate to difficulties with memory and concentration that some patients experience (5, 6). In the following sections, we explore the current state of exercise oncology, emphasizing how exercise is being integrated into cancer care protocols and highlighting the potential mechanisms through which physical activity exerts its beneficial effects. By providing healthcare professionals and researchers with a comprehensive overview of the latest insights and developments in this field, this review aims to foster a better understanding of the role of exercise in cancer care and encourage further research and clinical application of exercise as a standard component of oncological treatment strategies.

## 2 The positive impact of exercise on cancer prevention and treatment

**Figure 1:**
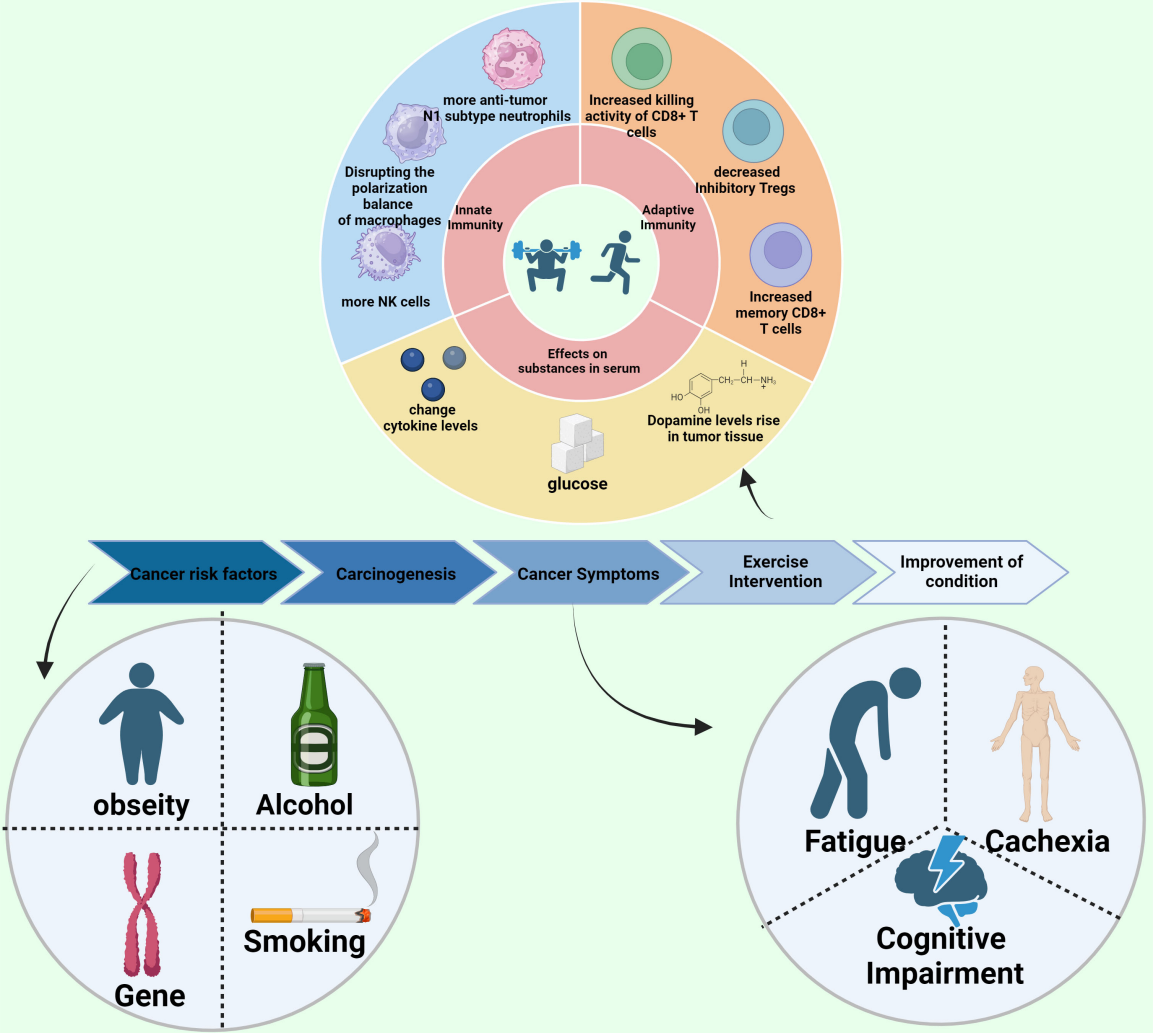
Exercise inhibits cancer. Schematic diagram of the review.

The prevailing view was once that cancer survivors should refrain from exercise, but contemporary research underscores that with meticulous supervision, they can engage in exercise regimens safely. Physical activity proves beneficial throughout the phases of cancer prevention, treatment, and survivorship (**Figure 1**).

### 2.1 Exercise reduces cancer incidence and prevents recurrence

To enhance the manuscript’s flow, discussions on exercise’s role in reducing the incidence and recurrence of cancer are consolidated. Exercise through its multiple forms such as aerobic and resistance activities significantly lowers the risk of developing cancer. This effect is supported by numerous studies including systematic reviews and meta-analyses demonstrating reduced risks for cancers such as breast, colon, and prostate, influencing body weight, inflammation levels, and hormonal balances. Transitioning from prevention, the subsequent sections will explore how exercise also amplifies the therapeutic efficacy of cancer treatments, thus providing a dual benefit in oncology care. These exercises also modulate various metabolic pathways that are often implicated in cancer progression (7, 8). For example, a landmark study demonstrated that regular exercise reduces the risk of colon cancer by up to 24% compared to individuals who are inactive (9). These findings have helped shape current exercise guidelines for cancer prevention, which recommend at least 150 minutes of moderate-intensity or 75 minutes of vigorous-intensity aerobic physical activity per week (10).

### 2.2 Exercise enhances the efficacy of cancer treatments

Cancer patients typically undergo various treatments, including surgery, chemotherapy, radiotherapy, targeted therapy, and immunotherapy. There is ample evidence that exercise enhances the effectiveness of these treatments. It also improves patient outcomes.

#### 2.2.1 Surgery

Surgery is a principal method for treating cancer, often utilized in clinical settings. However, surgical stress can induce significant acute systemic disturbances and local damage, which may lead to complications and promote cancer recurrence and metastasis through neuroendocrine, immune, and metabolic imbalances (10, 11). Clinical practice recognizes high cardiovascular reserve capacity and robust diastolic function as positive predictors for surgical outcomes (12). Preoperative exercise benefits include enhanced physical fitness, improved myocardial diastolic capacity, augmented contractile reserve, increased muscle mass, and reduced pulmonary congestion (13, 14). These benefits contribute to improved preoperative health, accelerated postoperative recovery, and reduced hospitalization duration (11). Research shows that postoperative rehabilitation training decreases complication rates post-gastric cancer surgery (15, 16), and home-based exercise programs are feasible for elderly cancer patients. There is a pressing need for large-scale, rigorous clinical trials to assess the role of exercise in surgical cancer treatment.

#### 2.2.2 Radiotherapy

Radiotherapy, utilized by approximately 60% of cancer patients, targets malignant tumors effectively (17). The success of radiotherapy hinges on the oxygenation of tumor tissues since oxygen is essential for generating reactive oxygen species (ROS) that damage cancer cells (18). Smaller, well-vascularized tumors with minimal hypoxic cells respond better to radiotherapy, whereas larger, poorly vascularized tumors with central necrosis are less responsive (19). By normalizing tumor vasculature and improving blood flow and oxygen delivery, exercise reduces tumor hypoxia and enhances radiotherapy’s efficacy. Experimental studies, such as those using 4T1 breast cancer and MC38 colorectal cancer (CRC) models, have demonstrated that when exercise is combined with radiotherapy, there is a significant reduction in tumor growth and metastasis (20). Furthermore, exercise is thought to bolster the immunological effects of radiotherapy. Animal studies have shown that exercise increases the secretion of endothelin, boosts natural killer (NK) cell infiltration, and enhances the expression of NK cell receptors such as Klrk1 and Il2rβ, with resistance training showing particular efficacy in these enhancements (21). Moreover, a combination of resistance and aerobic exercises has been observed to synergistically amplify these anticancer effects. In clinical settings, implementing exercise routines during radiotherapy has shown promising results; for example, resistance training significantly lowered serum kynurenine (KYN) levels in breast cancer patients, suggesting a non-pharmacological approach to improve radiotherapy outcomes (23).

#### 2.2.3 Chemotherapy

Recent studies underscore the importance of incorporating exercise into the regimen of cancer patients undergoing chemotherapy. Exercise demonstrates significant benefits for these patients by countering the negative effects of cytotoxic drugs, which often increase systemic inflammation and local tissue damage. This is achieved by modulating inflammation markers such as interleukin-6 (IL-6) and tumor necrosis factor-alpha (TNF-α), thereby reducing treatment-related fatigue and enhancing overall physical well-being (24–26). Preclinical studies have shown that exercise promotes angiogenesis, normalizes tumor vasculature, and improves drug delivery efficacy, illustrating the potential for exercise to magnify the anticancer effects of chemotherapeutic agents such as gemcitabine and doxorubicin (27, 28). For example, enhanced blood perfusion facilitated by exercise in tumor areas has been shown to improve the efficacy of these drugs (29).

Clinical evidence supports these findings; for instance, studies involving pancreatic cancer patients who engaged in structured exercise programs during neoadjuvant chemotherapy observed improvements in tumor vascularization, which plays a crucial role in optimizing drug delivery and enhancing treatment efficacy (9). Additionally, exercise interventions before and after chemotherapy have been linked with significantly reduced risks of recurrence and mortality in breast cancer patients, showcasing the potential of exercise as a complementary therapy (9).

To integrate insights from animal studies with clinical trial results, we have now included comparative analyses and summary tables in our manuscript. These additions emphasize the translational potential of preclinical findings and spotlight areas where human trials could further explore the mechanistic bases of these exercise benefits. This integrated approach not only clarifies how exercise contributes to enhanced chemotherapeutic outcomes but also provides a blueprint for future research aimed at harnessing exercise as a standard adjunct in cancer treatment protocols.

#### 2.2.4 Immunotherapy

Radiotherapy, utilized by approximately 60% of cancer patients, effectively targets malignant tumors. The success of radiotherapy hinges crucially on the oxygenation of tumor tissues, as oxygen is essential for generating reactive oxygen species (ROS) that damage cancer cells (17, 18). Smaller, well-vascularized tumors with minimal hypoxic cells respond better to radiotherapy, whereas larger, poorly vascularized tumors with central necrosis are less responsive (19).

Exercise improves blood flow and oxygen delivery by normalizing tumor vasculature, which reduces tumor hypoxia and enhances the efficacy of radiotherapy. Experimental studies, such as those using 4T1 breast cancer and MC38 colorectal cancer (CRC) models, have demonstrated that when exercise is combined with radiotherapy, there is a significant reduction in tumor growth and metastasis (20). Furthermore, exercise is thought to bolster the immunological effects of radiotherapy. Animal studies have shown that exercise increases the secretion of endothelin, boosts natural killer (NK) cell infiltration, and enhances the expression of NK cell receptors such as Klrk1 and Il2rβ, with resistance training showing particular efficacy in these enhancements (21).

In response to the reviewer’s comments, we have critically discussed the methodologies of the cited studies and expanded our examination of how variations in exercise protocols—such as intensity, duration, and type of exercise—impact the outcomes. This discussion now considers whether these differences have been adequately addressed in the literature and how they might contribute to discrepancies in study results. For example, we contrast the effects of aerobic versus resistance training in various cancer models and patient populations, and we highlight the need for standardized exercise protocols to facilitate more consistent and comparable results across studies.

This refined focus not only aligns with clinical observations but also sets a stage for future research to standardize exercise protocols as adjunct therapy in radiotherapy, ensuring more robust and reproducible benefits across diverse patient demographics.

### 2.3 Physical activity helps to lessen cancer symptoms and treatment-related adverse effects

Cancer and its array of treatments often result in substantial psychological and physiological distress, undermining both productivity and overall quality of life. Exercise is recognized for its role in mitigating these adverse effects, helping to sustain the physical vitality and mental resilience of patients, thereby enhancing their overall well-being. Numerous clinical guidelines advocate for physical activity in cancer care, supporting patients in maintaining a life enriched with activities beyond their identity as patients (37).

#### 2.3.1 Fatigue

Cancer-related fatigue (CRF) is a common symptom experienced by patients following a cancer diagnosis or the start of treatment, differing from typical fatigue in that it is not alleviated simply by rest (38). Research underscores the effectiveness of exercise in reducing CRF. The American Society of Clinical Oncology (ASCO) advises cancer survivors to engage in 150 minutes of moderate aerobic exercise weekly, like brisk walking or swimming, supplemented by two to three strength-training sessions (37, 39, 40). This regimen, while seemingly modest, significantly diminishes the intensity of CRF. A comprehensive meta-analysis involving 11,525 participants across 113 trials highlighted that exercise outperforms pharmacological interventions in reducing fatigue during and post cancer therapy (38). Moreover, a clinical trial segmented pancreatic cancer patients into two cohorts—one receiving standard care and the other standard care plus a structured exercise program. Results indicated that the exercise group exhibited notable enhancements in physical function, quality of life, and clinical symptoms, thus affirming the role of physical activity in augmenting exercise capacity and overall health status (41).

#### 2.3.2 Cancer cachexia

Cancer cachexia (CC) is a multifaceted syndrome prevalent among cancer patients, characterized by significant metabolic changes leading to progressive weight loss, primarily due to skeletal muscle atrophy, sometimes accompanied by fat loss. This syndrome is propelled by an inflammatory response that drives insulin resistance, hyperlipidemia, and mitochondrial dysfunction, thus perpetuating a deteriorating cycle (42). Although nutritional support is critical, it alone is insufficient to reverse the progression of CC (44). CC is particularly common in individuals with lung, colorectal, and gastrointestinal cancers, affecting over 85% of such patients (45, 46). While pharmaceutical solutions are being explored, standardized treatments remain limited.

Physical exercise serves as an effective non-pharmacological intervention for CC, significantly enhancing the survival rates and quality of life for affected patients. Most patients undergoing cancer treatment report a reduction in muscle mass and strength (47). This loss is often attributed to circulating tumor-derived factors that promote muscle degradation. Furthermore, the secretion of inflammatory adipokines in cancer patients may exacerbate insulin resistance, while the accumulation of intramuscular fat can impede blood flow within muscles, further aggravating metabolic imbalances and promoting tumor growth. Research has demonstrated that tumor-derived substances like parathyroid hormone-related protein and myostatin are key contributors to muscle atrophy and weight loss (47–50). Exercise has been shown to effectively reverse these effects. For example, engaging in voluntary wheel running during chemotherapy not only prevents weight loss but also maintains lean body mass and muscle strength, counteracting treatment-induced anorexia (51).

Despite promising results, the need for large-scale clinical trials to validate the effectiveness of exercise in managing CC remains. A particular study demonstrated the feasibility of exercise training among advanced cancer patients, although it was limited by its statistical power. Such multimodal approaches, which combine exercise, nutritional support, and pharmacotherapy, appear promising in addressing the multifaceted challenges of CC (52). Moving forward, research should focus on elucidating the molecular mechanisms through which exercise mitigates muscle atrophy and enhances metabolic functions in CC, potentially offering new avenues for treatment and patient care (43).

#### 2.3.3 Cognitive impairment

The causes of cognitive impairment in cancer patients are diverse and complex. Beyond the direct cognitive decline due to brain metastases from certain cancers, a multitude of factors contribute to this condition. These include the stress and psychological impact of a cancer diagnosis, the systemic effects of the cancer itself, various treatments, and genetic predispositions. Intriguingly, some breast cancer patients demonstrate cognitive dysfunction even prior to receiving any treatment, suggesting that specific characteristics inherent to breast cancer may predispose individuals to cognitive impairments. The Apolipoprotein E4 (APOE) gene, a well-documented risk factor for Alzheimer’s disease, has also been implicated in this context (53, 54). A prospective cohort study involving 943 breast cancer patients revealed that those adhering to national physical activity guidelines exhibited superior cognitive function both six months before and after undergoing chemotherapy, compared to their less active counterparts (55). Nonetheless, other studies report no significant correlation between higher self-reported physical activity levels during chemotherapy or follow-up and improved cognitive outcomes (56), highlighting the complexity of factors influencing cognitive health in cancer patients.

Larger clinical trials are underway to assess whether exercise can ameliorate cognitive impairment in cancer patients, focusing also on the underlying molecular mechanisms involved. These studies are designed to refine exercise protocols with the goal of enhancing cognitive functions, thereby improving the quality of life for those affected by cancer. By identifying the specific impacts of various forms of exercise on cognitive health, researchers hope to develop targeted strategies that effectively combat cognitive decline and boost overall mental capabilities in cancer patients. This approach could lead to more personalized exercise recommendations, tailored to the needs and health statuses of individual patients, maximizing the therapeutic benefits of physical activity in oncology settings.

## 3 Effects of exercise on tumor microenvironment regulation and molecular mechanisms

### 3.1 Effects of exercise on tumor angiogenesis

Angiogenesis is crucial for the progression, spread, and treatment of cancer. It involves the formation of new blood vessels, which is essential for supplying nutrients to tumors and facilitating the spread of cancer cells (57). Additionally, the oxygen carried through these new vessels enhances tumor perfusion, helping to reduce the effects of tumor hypoxia (58). Many cancer therapies target angiogenesis, using inhibitors to prevent the formation of these blood vessels within tumors. Improving vascular conductivity can alleviate tumor hypoxia, enhance the infiltration of immune cells into tumor tissues, inhibit tumor growth, and increase the effectiveness of cancer therapies (59).

However, the blood vessels in tumors are often abnormal and underdeveloped, leading to insufficient oxygen transport and significant tumor hypoxia (60). This hypoxic environment activates the production of hypoxia-inducible factor 1-α (HIF1α), which not only suppresses anti-cancer immune responses but also promotes cancer invasion and metastasis (57). Additionally, HIF1α drives abnormal angiogenesis, further deteriorating blood perfusion and oxygenation, thus reinforcing a cycle of hypoxia and increasingly aggressive cancer behaviors. Under these low-oxygen conditions, glycolysis is enhanced, resulting in an accumulation of lactate that negatively impacts the tumor’s immune microenvironment.

Exercise influences tumor angiogenesis, or the process by which new blood vessels form to supply the tumor, through several key mechanisms. Think of it as building new roads to improve the delivery of goods to a city—except in this case, the ‘goods’ are nutrients that unfortunately help the tumor grow. Firstly, it enhances the density and maturity of blood vessels within tumor tissues, promoting vascular normalization. This helps improve the delivery of oxygen and nutrients, which can affect tumor growth and treatment response. Secondly, exercise increases interaction between endothelial cells and other cells, boosting pericyte coverage and enhancing the expression of angiogenic factors like VEGFA in endothelial cells (61–63). Thirdly, it enhances nitric oxide utilization, a promoter of angiogenesis, by upregulating endothelial nitric oxide synthase, improving blood flow and oxygen delivery to tumor areas (62, 64). Additionally, exercise-induced secretion of myokines from skeletal muscles and adipokines from fat tissue plays a crucial role in angiogenesis (65, 66). Within the tumor immune microenvironment, exercise can decrease the number of M2 tumor-associated macrophages (TAMs) and prevent tumor-associated neutrophils (TANs) from shifting to a pro-angiogenic phenotype, thereby reducing the support for tumor growth and spread.

Alleviating tumor hypoxia and increasing oxygen levels can independently exert anti-cancer effects. An animal study showed that inhaling high concentrations of oxygen reduced tumor metastasis and improved survival rates (67). Enhanced oxygen supply can boost the efficacy of immune cells in attacking tumor cells by elevating pro-inflammatory cytokines and reducing immunosuppressive molecules and regulatory T cells in mouse tumors (67). However, further research is needed to fully understand how exercise impacts tumor vasculature in cancer patients. A notable study involving potential cancer patients demonstrated that exercise could significantly remodel human tumor vasculature. Moderate aerobic or anaerobic training increased both the number and density of blood vessels within tumor tissues, providing new avenues for targeting anti-cancer drugs more effectively through the enhanced vasculature (28).

### 3.2 Effects of exercise on cytokines and growth factors

The tumor microenvironment (TME) is a critical factor in cancer progression and treatment response. Exercise exerts a multifaceted impact on the TME through various mechanisms that influence tumor growth, immune responses, and overall disease trajectory. This section explores how physical activity compares with other non-pharmacological interventions, highlighting its unique contributions to cancer care. Exercise enhances the regulation of the TME primarily by improving systemic metabolism and reducing inflammation. Regular physical activity leads to an increased infiltration of immune cells into the tumor, enhancing anti-tumor immune responses (20). It also affects the production of cytokines and growth factors that can either inhibit or promote tumor growth, depending on their balance and the context of their release (21, 22). Comparatively, dietary modifications can also influence the TME but typically focus more on altering the nutrient supply to tumors and modifying systemic metabolic pathways that cancer cells exploit for growth and survival (23, 24). For example, ketogenic diets have been shown to reduce glucose availability to tumors, potentially slowing their growth. Psychological support, another crucial non-pharmacological intervention, primarily affects cancer outcomes by improving patients’ mental health, which can indirectly influence the TME by reducing stress-induced alterations in immune function and hormone levels (25). Stress reduction has been shown to decrease the production of pro-inflammatory cytokines and stress hormones that can promote tumor growth and metastasis (26). The integration of exercise with dietary changes and psychological support can provide a comprehensive approach that maximizes the therapeutic potential of each modality. While exercise directly modifies the physical and immune landscape of the TME, dietary interventions can starve tumors of necessary nutrients, and psychological support can maintain a healthier systemic environment less conducive to cancer progression (68). Future research should focus on creating integrated treatment protocols that combine these non-pharmacological interventions to optimize cancer treatment outcomes. By doing so, it is possible to leverage the unique advantages of exercise alongside dietary and psychological interventions, creating a multi-faceted strategy that addresses the complex nature of cancer and enhances patient quality of life.

#### 3.2.1 Myokines

Myokines (proteins released by muscle cells during exercise that have various biological effects) are a group of proteins and peptides secreted by skeletal muscles during exercise, including IL-6, irisin, decorin, IL-15, BDNF, IL-10, and IL-8. These can also be released from other organs and tissues. Myokines play dual roles in cancer biology, exhibiting either anti-tumor or pro-tumor activities depending on their nature and the surrounding environment. For instance, irisin can directly curtail tumor growth by inducing G2/M cell cycle arrest, escalating p21 levels, and simultaneously inhibiting cell proliferation and migration, while promoting apoptosis in glioblastoma cells (66, 69). Other myokines like IL-6 and IL-15 contribute to tumor suppression by hindering adipogenesis, while IL-6, IL-10, and IL-8 can bolster immune cell activity, enhancing their numbers and cytotoxic capabilities, thereby fostering a “hot” immune microenvironment conducive to fighting cancer.

IL-15, a prevalent myokine in skeletal muscle, is particularly important for its role in immunoregulation, supporting the proliferation and maturation of T cells and NK cells, crucial for the body’s defense against malignancies. Exercise stimulates the release of adrenaline, which can trigger a cascade leading to an acute anti-inflammatory/immunoregulatory state, resulting in the production of myokines such as IL-6 and IL-8. These myokines are essential for modulating NK cells and CD8+ T cells to combat tumor growth (36). Research has shown that exercise-induced IL-6 possesses both direct and indirect anti-cancer properties. For example, serum collected immediately after moderate-intensity aerobic interval exercise from men with lifestyle risk factors significantly reduced the proliferation of human colorectal cancer (CRC) cells, hinting at the potent inhibitory influence of IL-6 on these cells. This suppression of CRC cell proliferation by exercise could be partially attributed to IL-6-driven DNA damage and repair dynamics. Animal studies have illustrated that adrenaline and IL-6 released during exercise facilitate NK cell mobilization, redistribution, activation, and enhanced infiltration into tumor sites. Additionally, IL-6 might also alleviate cancer-related fatigue, potentially via the actions of pro-inflammatory cytokines IL-1β and TNF-α.

#### 3.2.2 Adipokines

Exercise influences adipogenesis and the metabolism of adipose tissue, with adipokines—proteins secreted by fat cells—having their secretion levels modified by physical activity. Dysregulated adipogenesis is a key contributor to cancer progression. Adipokines such as leptin, resistin, estrogen, macrophage migration inhibitory factor (MIF), and monocyte chemoattractant protein-1 (MCP-1) are instrumental in this context (70, 71). Leptin, for instance, promotes the growth of breast cancer cells, tumor angiogenesis, and inhibits apoptosis, whereas adiponectin exhibits opposing effects by reducing tumor cell proliferation and angiogenesis, thus restricting nutrient supply to tumors (72). Lower levels of adiponectin have been noted in patients with various cancers, including endometrial, esophageal, and liver cancers (65). Most other adipokines tend to facilitate cancer progression and metastasis by enhancing cell proliferation and migration, inhibiting apoptosis, and fostering inflammation.

A recently identified adipokine, kisspeptin, enhances the sensitivity of organs to glucose, lipids, and oxygen, thereby augmenting fat utilization during exercise and maintaining a balance between fat production and consumption (73). Exercise modulates adipose tissue by affecting adipokine levels, reducing adipogenesis, enhancing lipolysis, increasing glucose uptake and insulin sensitivity, and facilitating the conversion of white adipose tissue to brown adipose tissue (66, 74, 75). Studies, such as one involving a high-risk breast cancer population, have demonstrated that aerobic exercise training can reduce breast cancer risk by lowering body fat and modulating levels of leptin and adiponectin (76).

### 3.3 Effects of exercise on nutritional components and metabolism in cancer patients

#### 3.3.1 Nutritional components and metabolic pathways in cancer

Emerging research highlights the intricate relationship between nutrition, exercise, and cancer recovery, demonstrating how these elements interact to significantly influence patient outcomes. Studies involving post-surgical rats have underscored this interaction, revealing a notable decline in muscle strength and mass linked to the upregulation of genes associated with the ubiquitin-proteasome system, autophagy-lysosome system, and fast-twitch muscle fibers (77). Despite moderate exercise and amino acid supplementation, these rats exhibited reduced muscle strength. However, their gastrocnemius muscle mass increased, muscle atrophy was slowed, and genes related to fast-twitch fibers were downregulated, suggesting that postoperative intravenous amino acid and calcium ion supplementation combined with moderate exercise may help mitigate muscle loss.

Prolonged inactivity can disrupt the body’s nutrient balance, affecting crucial elements like glucose and calcium ions, as well as growth factors. Elevated levels of certain growth factors can activate significant metabolic pathways such as the PI3K/AKT/mTOR pathway, enhancing nutrient absorption and utilization, potentially facilitating tumor growth and progression (78). Regular, long-term exercise has been shown to reduce the levels of these growth factors in the bloodstream, improve overall metabolic rates, and decrease the stimulation of cancerous tissues by these growth factors. Chronic physical activity substantially increases glucose uptake by skeletal muscles, reduces circulating glucose levels, and decreases both insulin and insulin-like growth factor (IGF) concentrations (79).

The general effects of exercise on growth factors, cytokines, nutrients, and metabolites are well-documented. However, more targeted clinical and basic research is required to verify these impacts specifically in the tumor tissues of cancer patients. For instance, a study involving prostate cancer patients who underwent a 12-week exercise program revealed increased serum levels of oncostatin M and myokines, decreased IGF levels, and a slowdown in tumor cell growth, supporting the potential tumor-inhibitory effects of exercise (80). Post-treatment exercise interventions have also shown beneficial impacts on IGF1 and inflammatory biomarkers in breast cancer patients (81). Additionally, research on breast cancer survivors demonstrated that a combined regimen of aerobic and anaerobic exercises effectively ameliorated metabolic disorders, reduced circulating biomarkers related to insulin resistance and inflammatory responses—such as insulin, IGF-1, IL-6, IL-8, and TNF-α—and significantly decreased endothelin levels, which are associated with muscle loss and degeneration. Concurrently, these exercises increased adiponectin levels, further illustrating the multifaceted benefits of physical activity in managing cancer-related metabolic disruptions (80, 82).

### 3.4 Effects of exercise on anti-cancer immunity

Exercise plays a pivotal role in modulating the immune landscape within cancer patients, impacting both innate and adaptive immune responses. This section delves into how exercise influences these responses and highlights the potential for personalizing exercise regimens to enhance their efficacy based on individual patient profiles.

Physical activity has been shown to significantly improve the functionality and number of various immune cells, which are crucial for combatting cancer. For example, exercise boosts the number and activity of natural killer (NK) cells, T cells, and dendritic cells, all of which play roles in recognizing and destroying cancer cells (30, 31). Regular exercise also reduces systemic inflammation, a common contributor to immunosuppression in cancer patients, thereby enhancing the overall immune surveillance and response to tumors (32).

Personalizing exercise regimens can maximize these immunological benefits by tailoring the intensity, duration, and type of exercise to individual patient needs. Factors such as the patient’s cancer type, treatment stage, overall health, and genetic makeup should guide the customization of exercise programs. For instance, patients with solid tumors might benefit more from moderate-intensity aerobic exercises, which have been shown to improve blood flow and oxygenation to the tumor site, enhancing the efficacy of other treatments like chemotherapy and radiotherapy (33).

Additionally, understanding the genetic and metabolic profiles of cancer patients can further refine exercise prescriptions. Genetic markers related to inflammation and immune cell functionality, such as variations in cytokine genes, can indicate how a patient might respond to different forms of exercise (34). Similarly, metabolic profiling can reveal how exercise could influence cancer metabolism directly or support the body’s natural anti-cancer mechanisms (35).

Ongoing research is increasingly supporting the idea of integrating biomarker analysis into routine clinical practice to guide exercise recommendations. By assessing markers of inflammation, immune cell activity, and metabolic function, clinicians can develop more effective, personalized exercise plans that not only support the patient’s general health but also directly contribute to cancer treatment and recovery.

Future studies should focus on longitudinal analyses to better understand the long-term effects of personalized exercise on cancer prognosis. Such research will provide deeper insights into the optimal exercise modalities for different cancer types and stages, potentially leading to standardized yet customizable exercise guidelines within oncology.

#### 3.4.1 Innate immunity

Natural killer (NK) cells are essential players in the body’s innate immune response, and their activity and numbers can be significantly influenced by exercise. Research using mouse tumor models has demonstrated that interventions such as wheel-running increase NK cell infiltration into tumor tissues, which considerably slows cancer growth. This effect is primarily mediated by adrenaline and muscle-derived interleukin-6 (IL-6) (48, 83). Although exercise does not directly enhance the cytotoxicity of NK cells, it upregulates ligands for activating NK cell receptors in both mouse cancer models and human studies, thereby enhancing their cytotoxic potential (48, 84). Furthermore, combining exercise with radiation therapy has shown to increase NK cell infiltration and upregulate gene expression of NK cell receptors, boosting the effectiveness of the radiation treatment (21, 85).

Macrophages also play a crucial role in innate immunity and anti-cancer responses, with the pro-inflammatory M1 phenotype exhibiting anti-tumor effects, while the anti-inflammatory M2 phenotype supports tumor growth by releasing factors like IL-10 and TGF-β. Exercise can influence macrophage polarization towards the M1 phenotype, enhancing anti-cancer effects. Studies suggest that long-term exercise disrupts the balance of macrophage polarization, increasing differentiation towards the M1 phenotype, and thus contributing to the delay in cancer progression (86). However, the detailed mechanisms through which exercise influences macrophage activity remain largely unexplored.

Neutrophils, particularly tumor-associated neutrophils (TANs), play dual roles in cancer progression. The pro-tumor N2 subtype and the anti-tumor N1 subtype of TANs directly and indirectly regulate cancer cell survival, migration, immune function, and angiogenesis (87). Preclinical studies indicate that both swimming and running can significantly delay tumor growth, associated with a reduction in neutrophil counts (88–90). Furthermore, exercise-induced release of high-mobility group box 1 (HMGB1) has been observed to enhance citric acid metabolism in the tricarboxylic acid cycle, thereby improving immunosurveillance of senescent cells in a mechanism dependent on nuclear factor erythroid 2–related factor 2 (NRF2) (11). These findings underline the significant role of exercise in modulating the innate immune response against cancer, suggesting potential therapeutic benefits for cancer patients.

#### 3.4.2 Adaptive immunity

Exercise has demonstrated a positive influence on adaptive anti-cancer immunity as well. In various studies, particularly with mouse models of breast cancer, physical activity has been shown to not only increase the number of CD8+ T cells infiltrating tumors but also to enhance their cytotoxic capabilities. This boost in CD8+ T cell activity due to exercise may be mediated through the CXCL9/11-CXCR3 signaling pathway, which is crucial for T cell recruitment and function (91). Another research finding suggests that exercise improves CD8+ T cell efficacy by altering central carbon metabolism, thus optimizing their energy use and functional capacity (92).

In models of pancreatic cancer, exercise has been found to facilitate the mobilization and intra-tumoral clustering of IL15Rα+ CD8+ T cells, thereby amplifying the anti-tumor immune responses (93). Importantly, the augmentation in CD8+ T cells due to regular physical activity can significantly enhance the effectiveness of standard anti-cancer treatments, such as immunotherapy and radiotherapy (85, 91, 93).

Moreover, exercise impacts adaptive immunity by regulating various factors that not only increase the infiltration of CD8+ T cells into tumors but also boost their expression of functional molecules, crucial for their anti-tumor activity. Concurrently, exercise has been observed to decrease the presence of immunosuppressive regulatory T cells (Tregs), which can otherwise hinder effective immune responses against tumors. Additionally, physical activity appears to increase the number of memory CD8+ T cells, which are important for long-term immune surveillance and cancer control.

These findings indicate that regular exercise can potentiate the anti-cancer efficacy of treatments like radiotherapy and therapies targeting PD-(L)1, by modulating the immune landscape in favor of a more robust anti-tumor response. This highlights the potential of exercise as a strategic complement in cancer treatment protocols to leverage the body’s own immune system against cancer.

### 3.5 Effects of exercise on cancer cells

Basic research has highlighted that exercise can impact cancer cells by hindering their proliferation, promoting apoptosis, and reducing their migration capabilities. For example, in a study involving colorectal cancer (CRC) patients, it was found that serum altered by exercise significantly suppressed the proliferation of LOVO cancer cells. Acute exercise leads to a rise in serum IL-6 levels (94), which in turn stimulates the release of anti-inflammatory cytokines, mobilizes immune cells, and helps mitigate DNA damage in early malignant cells, producing a range of beneficial biological effects. *In vitro* experiments further demonstrated that recombinant IL-6 at concentrations of 10 and 100 pg/mL could inhibit human CRC cell proliferation and reduce γ-H2AX expression, reflecting the anti-cancer properties associated with exercise. Additionally, recent research has shown that serum from metastatic castration-resistant prostate cancer (mCRPC) patients, who engaged in long-term regular exercise, exhibited delayed proliferation of human prostate cancer cells (95).

The mechanisms through which exercise influences cancer cells are complex and multifaceted. Firstly, exercise reduces levels of various nutrients and growth factors, such as glucose and insulin-like growth factors (IGFs), which are known to activate key pro-cancer signaling pathways like the PI3K/Akt/mTOR pathway (96, 97). At the same time, it activates anti-cancer signaling pathways, such as the AMPK pathway (98). Secondly, exercise affects cancer biology by altering the levels of critical growth factors and cytokines secreted by other organs. For instance, exercise-induced myokines like IL-10 and CCL4 have been shown to directly reduce tumor cell growth and migration in pancreatic cancer patients (99). Thirdly, exercise has been observed to suppress the Hippo/YAP signaling pathway in cancer cells, thereby inhibiting tumor formation and cell viability (100). Furthermore, moderate exercise increases dopamine levels in tumor tissues, which helps inhibit cancer cell growth and lung metastasis through mechanisms dependent on dopamine receptor 2 and TGF-β1 (101).

## 4 Conclusion and future perspectives

This review confirms the significant anti-cancer benefits of exercise, including reducing tumor incidence, suppressing tumor growth, mitigating treatment-related side effects, and enhancing overall survival rates. Such benefits underscore the necessity of integrating exercise as a standard component of cancer care protocols across all stages of the disease.

Future research should focus on elucidating the specific molecular and cellular mechanisms by which exercise impacts cancer, which will aid in developing targeted therapeutic strategies that leverage exercise’s full potential (102). A deeper understanding of these mechanisms is essential for optimizing the design of exercise programs that can be tailored to individual needs based on cancer type, stage, and patient-specific characteristics such as genetic, metabolic, and immunological profiles.

There is a compelling need for personalized exercise prescriptions to maximize the therapeutic potential of exercise in oncology (103, 104). These prescriptions should be crafted by interdisciplinary teams, including oncologists, exercise physiologists, and data scientists, to ensure that exercise interventions are safe, effective, and specifically tailored to individual patient demographics. Additionally, it is crucial to address potential risks associated with exercise, particularly for patients with advanced cancer or significant comorbidities, by developing comprehensive guidelines that ensure exercise programs are implemented safely.

Enhancing cooperation among various healthcare professionals is vital for developing more effective exercise programs tailored to the specific needs of cancer patients (105). This collaborative approach can help overcome barriers to the implementation of exercise as a therapeutic strategy and pave the way for more inclusive, holistic cancer treatment plans.

Moreover, longitudinal studies are needed to better understand the long-term effects of exercise on cancer recurrence and survival. These studies will help establish robust, evidence-based guidelines for incorporating physical activity into cancer recovery and long-term survivorship plans (106, 107). Such research is essential for substantiating the benefits of exercise in the oncology setting and for encouraging its broader adoption in routine clinical practice.

Ultimately, these efforts will better harness the potential of exercise to complement traditional cancer therapies, potentially transforming the standard of care in oncology and markedly improving patient outcomes. By advancing our understanding and integration of exercise in cancer treatment, we can hope to significantly enhance the quality of life and survival rates for cancer patients worldwide.

## References

[1] Koelwyn GJ Quail DF Zhang X White RM Jones LW. Exercise-dependent regulation of the tumour microenvironment. Nat Rev Cancer. (2017) 17:620–32. doi: 10.1038/nrc.2017.78 28943640

[2] Sarich P Canfell K Egger S Banks E Joshy G Grogan P. Alcohol consumption, drinking patterns and cancer incidence in an Australian cohort of 226,162 participants aged 45 years and over. Br J Cancer. (2021) 124:513–23. doi: 10.1038/s41416-020-01101-2 PMC7853127 33041337

[3] Sohn W Lee HW Lee S Lim JH Lee MW Park CH. Obesity and the risk of primary liver cancer: A systematic review and meta-analysis. Clin Mol Hepatol. (2021) 27:157–74. doi: 10.3350/cmh.2020.0176 PMC7820201 33238333

[4] Matthews CE Moore SC Arem H Cook MB Trabert B Håkansson N. Amount and intensity of leisure-time physical activity and lower cancer risk. J Clin Oncol. (2020) 38:686–97. doi: 10.1200/JCO.19.02407 PMC7048166 31877085

[5] Hojman P Gehl J Christensen JF Pedersen BK. Molecular mechanisms linking exercise to cancer prevention and treatment. Cell Metab. (2018) 27:10–21. doi: 10.1016/j.cmet.2017.09.015 29056514

[6] Fiuza-Luces C Valenzuela PL Gálvez BG Ramírez M López-Soto A Simpson RJ. The effect of physical exercise on anticancer immunity. Nat Rev Immunol. (2024) 24:282–93. doi: 10.1038/s41577-023-00943-0 37794239

[7] Schmitz KH Campbell AM Stuiver MM Pinto BM Schwartz AL Morris GS. Exercise is medicine in oncology: Engaging clinicians to help patients move through cancer. CA Cancer J Clin. (2019) 69:468–84. doi: 10.3322/caac.21579 PMC7896280 31617590

[8] Mctiernan A Friedenreich CM Katzmarzyk PT Powell KE Macko R Buchner D. Physical activity in cancer prevention and survival: A systematic review. Med Sci Sports Exerc. (2019) 51:1252–61. doi: 10.1249/MSS.0000000000001937 PMC6527123 31095082

[9] Cannioto RA Hutson A Dighe S McCann W McCann SE Zirpoli GR. Physical activity before, during, and after chemotherapy for high-risk breast cancer: relationships with survival. JNCI J Natl Cancer Inst. (2021) 113:54–63. doi: 10.1093/jnci/djaa046 32239145 PMC7781460

[10] Tohme S Simmons RL Tsung A. Surgery for cancer: A trigger for metastases. Cancer Res. (2017) 77:1548–52. doi: 10.1158/0008-5472.CAN-16-1536 PMC5380551 28330928

[11] Zhang H Chen T Ren J Xia Y Onuma A Wang Y. Pre-operative exercise therapy triggers anti-inflammatory trained immunity of Kupffer cells through metabolic reprogramming. Nat Metab. (2021) 3:843–58. doi: 10.1038/s42255-021-00402-x PMC8462058 34127858

[12] Gulsin GS Henson J Brady EM Sargeant JA Wilmot EG Athithan L. Cardiovascular determinants of aerobic exercise capacity in adults with type 2 diabetes. Diabetes Care. (2020) 43:2248–56. doi: 10.2337/dc20-0706 PMC7440912 32680830

[13] McIsaac DI Hladkowicz E Bryson GL Forster AJ Gagne S Huang A. Home-based prehabilitation with exercise to improve postoperative recovery for older adults with frailty having cancer surgery: the PREHAB randomised clinical trial. Br J Anaesth. (2022) 129:41–8. doi: 10.1016/j.bja.2022.04.006 35589429

[14] Roh JD Houstis N Yu A Chang B Yeri A Li H. Exercise training reverses cardiac aging phenotypes associated with heart failure with preserved ejection fraction in male mice. Aging Cell. (2020) 19:e13159. doi: 10.1111/acel.13159 32441410 PMC7294786

[15] Bausys A Luksta M Anglickiene G Maneikiene VV Kryzauskas M Rybakovas A. Effect of home-based prehabilitation on postoperative complications after surgery for gastric cancer: randomized clinical trial. Br J Surg. (2023) 110:1800–7. doi: 10.1093/bjs/znad312 37750588

[16] Barnes K Hladkowicz E Dorrance K Bryson GL Forster AJ Gagné S. Barriers and facilitators to participation in exercise prehabilitation before cancer surgery for older adults with frailty: a qualitative study. BMC Geriatr. (2023) 23:356. doi: 10.1186/s12877-023-03990-3 37280523 PMC10242997

[17] Morris ZS Harari PM. Interaction of radiation therapy with molecular targeted agents. J Clin Oncol. (2014) 32:2886–93. doi: 10.1200/JCO.2014.55.1366 PMC4152717 25113770

[18] Chen HHW Kuo MT. Improving radiotherapy in cancer treatment: Promises and challenges. Oncotarget. (2017) 8:62742–58. doi: 10.18632/oncotarget.18409 PMC5617545 28977985

[19] Telarovic I Wenger RH Pruschy M. Interfering with tumor hypoxia for radiotherapy optimization. J Exp Clin Cancer Res. (2021) 40:197. doi: 10.1186/s13046-021-02000-x 34154610 PMC8215813

[20] Ashcraft KA Warner AB Jones LW Dewhirst MW. Exercise as adjunct therapy in cancer. Semin Radiat Oncol. (2019) 29:16–24. doi: 10.1016/j.semradonc.2018.10.001 30573180 PMC6656408

[21] Dufresne S Guéritat J Chiavassa S Noblet C Assi M Rioux-Leclercq N. Exercise training improves radiotherapy efficiency in a murine model of prostate cancer. FASEB J Off Publ Fed Am Soc Exp Biol. (2020) 34:4984–96. doi: 10.1096/fj.201901728R 32043634

[22] Vikmoen O Strandberg E Svindland KV Henriksson A Mazzoni A-S Johansson B. Effects of heavy-load strength training during (neo-)adjuvant chemotherapy on muscle strength, muscle fiber size, myonuclei, and satellite cells in women with breast cancer. FASEB J Off Publ Fed Am Soc Exp Biol. (2024) 38:e23784. doi: 10.1096/fj.202400634R 38953567

[23] Zimmer P Schmidt ME Prentzell MT Berdel B Wiskemann J Kellner KH. Resistance exercise reduces kynurenine pathway metabolites in breast cancer patients undergoing radiotherapy. Front Oncol. (2019) 9:962. doi: 10.3389/fonc.2019.00962 31612110 PMC6773833

[24] Gouez M Rébillard A Thomas A Beaumel S Matera E-L Gouraud E. Combined effects of exercise and immuno-chemotherapy treatments on tumor growth in MC38 colorectal cancer-bearing mice. Front Immunol. (2024) 15:1368550. doi: 10.3389/fimmu.2024.1368550 38426110 PMC10902641

[25] Hiensch AE Mijwel S Bargiela D Wengström Y May AM Rundqvist H. Inflammation mediates exercise effects on fatigue in patients with breast cancer. Med Sci Sports Exerc. (2021) 53:496–504. doi: 10.1249/MSS.0000000000002490 32910094 PMC7886356

[26] Neuzillet C Bouché O Tournigand C Chibaudel B Bauguion L Bengrine-Lefevre L. Effect of adapted physical activity in patients with advanced pancreatic cancer: the APACaP GERCOR randomized trial. J Natl Compr Cancer Netw JNCCN. (2023) 21:1234–1242.e17. doi: 10.6004/jnccn.2023.7065 38081120

[27] Wakefield ZR Tanaka M Pampo C Lepler S Rice L Guingab-Cagmat J. Normal tissue and tumor microenvironment adaptations to aerobic exercise enhance doxorubicin anti-tumor efficacy and ameliorate its cardiotoxicity in retired breeder mice. Oncotarget. (2021) 12:1737–48. doi: 10.18632/oncotarget.28057 PMC8416558 34504647

[28] Florez Bedoya CA Cardoso ACF Parker N Ngo-Huang A Petzel MQ Kim MP. Exercise during preoperative therapy increases tumor vascularity in pancreatic tumor patients. Sci Rep. (2019) 9:13966. doi: 10.1038/s41598-019-49582-3 31562341 PMC6765012

[29] Schauer T Mazzoni A-S Henriksson A Demmelmaier I Berntsen S Raastad T. Exercise intensity and markers of inflammation during and after (neo-) adjuvant cancer treatment. Endocr Relat Cancer. (2021) 28:191–201. doi: 10.1530/ERC-20-0507 33608485

[30] Campbell KL Brown JC Lee C Weltzien E Li J Sternfeld B. Advances in adherence reporting of resistance training in a clinical trial during adjuvant chemotherapy for colon cancer. Med Sci Sports Exerc. (2024) 56:1186–95. doi: 10.1249/MSS.0000000000003395 PMC11096063 38233992

[31] Kirkham AA Gelmon KA Van Patten CL Bland KA Wollmann H McKenzie DC. Impact of exercise on chemotherapy tolerance and survival in early-stage breast cancer: A nonrandomized controlled trial. J Natl Compr Canc Netw. (2020) 18:1670–7. doi: 10.6004/jnccn.2020.7603 33285521

[32] Chiarotto JA Akbarali R Bellotti L Dranitsaris G. A structured group exercise program for patients with metastatic cancer receiving chemotherapy and CTNNB1 (&beta;-catenin) as a biomarker of exercise efficacy. Cancer Manag Res. (2017) 9:495–501. doi: 10.2147/CMAR.S147054 29075139 PMC5648300

[33] Groen WG Naaktgeboren WR van Harten WH van Vulpen JK Kool N Sonke GS. Physical fitness and chemotherapy tolerance in patients with early-stage breast cancer. Med Sci Sports Exerc. (2022) 54:537–42. doi: 10.1249/MSS.0000000000002828 PMC8920022 34961754

[34] Gustafson MP Wheatley-Guy CM Rosenthal AC Gastineau DA Katsanis E Johnson BD. Exercise and the immune system: taking steps to improve responses to cancer immunotherapy. J Immunother Cancer. (2021) 9:e001872. doi: 10.1136/jitc-2020-001872 34215686 PMC8256759

[35] Yan H Jiang A Huang Y Zhang J Yang W Zhang W. Exercise sensitizes PD-1/PD-L1 immunotherapy as a hypoxia modulator in the tumor microenvironment of melanoma. Front Immunol. (2023) 14:1265914. doi: 10.3389/fimmu.2023.1265914 37876940 PMC10590877

[36] Farley MJ Bartlett DB Skinner TL Schaumberg MA Jenkins DG. Immunomodulatory function of interleukin-15 and its role in exercise, immunotherapy, and cancer outcomes. Med Sci Sports Exerc. (2023) 55:558–68. doi: 10.1249/MSS.0000000000003067 36730979

[37] Stout NL Santa Mina D Lyons KD Robb K Silver JK. A systematic review of rehabilitation and exercise recommendations in oncology guidelines. CA Cancer J Clin. (2021) 71:149–75. doi: 10.3322/caac.21639 PMC7988887 33107982

[38] Mustian KM Alfano CM Heckler C Kleckner AS Kleckner IR Leach CR. Comparison of pharmaceutical, psychological, and exercise treatments for cancer-related fatigue: A meta-analysis. JAMA Oncol. (2017) 3:961. doi: 10.1001/jamaoncol.2016.6914 28253393 PMC5557289

[39] Wagoner CW Lee JT Battaglini CL. Community-based exercise programs and cancer-related fatigue: a systematic review and meta-analysis. Support Care Cancer Off J Multinatl Assoc Support Care Cancer. (2021) 29:4921–9. doi: 10.1007/s00520-021-06135-7 33751225

[40] Bower JE Bak K Berger A Breitbart W Escalante CP Ganz PA. Screening, assessment, and management of fatigue in adult survivors of cancer: an american society of clinical oncology clinical practice guideline adaptation. J Clin Oncol. (2014) 32:1840–50. doi: 10.1200/JCO.2013.53.4495 PMC4039870 24733803

[41] Ngo-Huang AT Parker NH Xiao L SChadler KL Petzel MQB Prakash LR. Effects of a pragmatic home-based exercise program concurrent with neoadjuvant therapy on physical function of patients with pancreatic cancer: the pancFit randomized clinical trial. Ann Surg. (2023) 278:22–30. doi: 10.1097/SLA.0000000000005878 37026453 PMC10330108

[42] Mavropalias G Sim M Taaffe DR Galvão DA Spry N Kraemer WJ. Exercise medicine for cancer cachexia: targeted exercise to counteract mechanisms and treatment side effects. J Cancer Res Clin Oncol. (2022) 148:1389–406. doi: 10.1007/s00432-022-03927-0 PMC9114058 35088134

[43] Hardee JP Counts BR Carson JA. Understanding the role of exercise in cancer cachexia therapy. Am J Lifestyle Med. (2019) 13:46–60. doi: 10.1177/1559827617725283 30627079 PMC6311610

[44] Franco FDO Lopes MA Henriques FDS Neves RXD Bianchi Filho C Batista ML. Cancer cachexia differentially regulates visceral adipose tissue turnover. J Endocrinol. (2017) 232:493–500. doi: 10.1530/JOE-16-0305 28053001

[45] Dodson S Baracos VE Jatoi A Evans WJ Cella D Dalton JT. Muscle wasting in cancer cachexia: clinical implications, diagnosis, and emerging treatment strategies. Annu Rev Med. (2011) 62:265–79. doi: 10.1146/annurev-med-061509-131248 20731602

[46] Leal LG Lopes MA Peres SB Batista ML. Exercise training as therapeutic approach in cancer cachexia: A review of potential anti-inflammatory effect on muscle wasting. Front Physiol. (2021) 11:570170. doi: 10.3389/fphys.2020.570170 33613297 PMC7890241

[47] Raun SH Buch-Larsen K Schwarz P Sylow L. Exercise-A panacea of metabolic dysregulation in cancer: physiological and molecular insights. Int J Mol Sci. (2021) 22:3469. doi: 10.3390/ijms22073469 33801684 PMC8037630

[48] Pedersen L Idorn M Olofsson GH Lauenborg B Nookaew I Hansen RH. Voluntary running suppresses tumor growth through epinephrine- and IL-6-dependent NK cell mobilization and redistribution. Cell Metab. (2016) 23:554–62. doi: 10.1016/j.cmet.2016.01.011 26895752

[49] Gallot YS Durieux A-C Castells J Desgeorges MM Vernus B Plantureux L. Myostatin gene inactivation prevents skeletal muscle wasting in cancer. Cancer Res. (2014) 74:7344–56. doi: 10.1158/0008-5472.CAN-14-0057 25336187

[50] Kir S White JP Kleiner S Kazak L Cohen P Baracos VE. Tumour-derived PTH-related protein triggers adipose tissue browning and cancer cachexia. Nature. (2014) 513:100–4. doi: 10.1038/nature13528 PMC4224962 25043053

[51] Barnes O Wilson RL Gonzalo-Encabo P Kang D-W Christopher CN Bentley T. The effect of exercise and nutritional interventions on body composition in patients with advanced or metastatic cancer: A systematic review. Nutrients. (2022) 14:2110. doi: 10.3390/nu14102110 35631251 PMC9145470

[52] Solheim TS Laird BJA Balstad TR Stene GB Bye A Johns N. A randomized phase II feasibility trial of a multimodal intervention for the management of cachexia in lung and pancreatic cancer. J Cachexia Sarcopenia Muscle. (2017) 8:778–88. doi: 10.1002/jcsm.12201 PMC5659068 28614627

[53] Mandelblatt JS Small BJ Luta G Hurria A Jim H McDonald BC. Cancer-related cognitive outcomes among older breast cancer survivors in the thinking and living with cancer study. J Clin Oncol. (2018) 36:3211–22. doi: 10.1200/JCO.18.00140 PMC7237199 30281396

[54] Speidell AP Demby T Lee Y Rodriguez O Albanese C Mandelblatt J. Development of a human APOE knock-in mouse model for study of cognitive function after cancer chemotherapy. Neurotox Res. (2019) 35:291–303. doi: 10.1007/s12640-018-9954-7 30284204 PMC6333492

[55] Salerno EA Culakova E Kleckner AS Heckler CE Lin P-J Matthews CE. Physical activity patterns and relationships with cognitive function in patients with breast cancer before, during, and after chemotherapy in a prospective, nationwide study. J Clin Oncol. (2021) 39:3283–92. doi: 10.1200/JCO.20.03514 PMC8500586 34406822

[56] Naaktgeboren WR Koevoets EW Stuiver MM van Harten WH Aaronson NK van der Wall E. Effects of physical exercise during adjuvant chemotherapy for breast cancer on long-term tested and perceived cognition: results of a pragmatic follow-up study. Breast Cancer Res Treat. (2024) 205:75–86. doi: 10.1007/s10549-023-07220-7 38285111 PMC11062992

[57] Lugano R Ramachandran M Dimberg A. Tumor angiogenesis: causes, consequences, challenges and opportunities. Cell Mol Life Sci. (2020) 77:1745–70. doi: 10.1007/s00018-019-03351-7 PMC7190605 31690961

[58] Esteves M Monteiro MP Duarte JA. The effects of physical exercise on tumor vasculature: systematic review and meta-analysis. Int J Sports Med. (2021) 42:1237–49. doi: 10.1055/a-1533-1876 34341974

[59] Esteves M Monteiro MP Duarte JA. Role of regular physical exercise in tumor vasculature: favorable modulator of tumor milieu. Int J Sports Med. (2021) 42:389–406. doi: 10.1055/a-1308-3476 33307553

[60] Bennewith KL Durand RE. Quantifying transient hypoxia in human tumor xenografts by flow cytometry. Cancer Res. (2004) 64:6183–9. doi: 10.1158/0008-5472.CAN-04-0289 15342403

[61] Betof AS Lascola CD Weitzel D Landon C Scarbrough PM Devi GR. Modulation of murine breast tumor vascularity, hypoxia, and chemotherapeutic response by exercise. JNCI J Natl Cancer Inst. (2015) 107(5):djv040. doi: 10.1093/jnci/djv040 25780062 PMC4822524

[62] Fiorenza M Gliemann L Brandt N Bangsbo J. Hormetic modulation of angiogenic factors by exercise-induced mechanical and metabolic stress in human skeletal muscle. Am J Physiol-Heart Circ Physiol. (2020) 319(4):H824–H834. doi: 10.1152/ajpheart.00432.2020 32822216

[63] SChadler KL Thomas NJ Galie PA Bhang DH Roby KC Addai P. Tumor vessel normalization after aerobic exercise enhances chemotherapeutic efficacy. Oncotarget. (2016) 7:65429–40. doi: 10.18632/oncotarget.11748 PMC5323166 27589843

[64] Gallo O Fini-Storchi I Vergari WA Masini E Morbidelli L Ziche M. Role of nitric oxide in angiogenesis and tumor progression in head and neck cancer. JNCI J Natl Cancer Inst. (1998) 90:587–96. doi: 10.1093/jnci/90.8.587 9554441

[65] Perego S Sansoni V Ziemann E Lombardi G. Another weapon against cancer and metastasis: physical-activity-dependent effects on adiposity and adipokines. Int J Mol Sci. (2021) 22:2005. doi: 10.3390/ijms22042005 33670492 PMC7922129

[66] Kim J-S Galvão DA Newton RU Gray E Taaffe DR. Exercise-induced myokines and their effect on prostate cancer. Nat Rev Urol. (2021) 18:519–42. doi: 10.1038/s41585-021-00476-y 34158658

[67] Hatfield SM Kjaergaard J Lukashev D Schreiber TH Belikoff B Abbott R. Immunological mechanisms of the antitumor effects of supplemental oxygenation. Sci Transl Med. (2015) 7(277):277ra30. doi: 10.1126/scitranslmed.aaa1260 PMC4641038 25739764

[68] Luo Z Wan R Liu S Feng X Peng Z Wang Q. Mechanisms of exercise in the treatment of lung cancer – a mini-review. Front Immunol. (2023) 14:1244764. doi: 10.3389/fimmu.2023.1244764 37691942 PMC10483406

[69] Huang C-W Chang Y-H Lee H-H Wu J-Y Huang J-X Chung Y-H. Irisin, an exercise myokine, potently suppresses tumor proliferation, invasion, and growth in glioma. FASEB J Off Publ Fed Am Soc Exp Biol. (2020) 34:9678–93. doi: 10.1096/fj.202000573RR 32469121

[70] Mannelli M Gamberi T Magherini F Fiaschi T. The adipokines in cancer cachexia. Int J Mol Sci. (2020) 21:4860. doi: 10.3390/ijms21144860 32660156 PMC7402301

[71] Pu X Chen D. Targeting adipokines in obesity-related tumors. Front Oncol. (2021) 11:685923. doi: 10.3389/fonc.2021.685923 34485124 PMC8415167

[72] Vona-Davis L Rose DP. Adipokines as endocrine, paracrine, and autocrine factors in breast cancer risk and progression. Endocr Relat Cancer. (2007) 14:189–206. doi: 10.1677/ERC-06-0068 17639037

[73] LiangCLiXSongGSchmidtSFSunLChenJ. Adipose Kiss1 controls aerobic exercise-related adaptive responses in adipose tissue energy homeostasis. FASEB J Off Publ Fed Am Soc Exp Biol. (2024) 38:e23743. doi: 10.1096/fj.202302598RR 38877852

[74] Supriya R Delfan M Saeidi A Samaie SS Al Kiyumi MH Escobar KA. Spirulina supplementation with high-intensity interval training decreases adipokines levels and cardiovascular risk factors in men with obesity. Nutrients. (2023) 15:4891. doi: 10.3390/nu15234891 38068748 PMC10707917

[75] Golbidi S Laher I. Exercise induced adipokine changes and the metabolic syndrome. J Diabetes Res. (2014) 2014:1–16. doi: 10.1155/2014/726861 PMC3915640 24563869

[76] Sturgeon K Digiovanni L Good J Salvatore D Fenderson D Domchek S. Exercise-induced dose-response alterations in adiponectin and leptin levels are dependent on body fat changes in women at risk for breast cancer. Cancer Epidemiol Biomarkers Prev. (2016) 25:1195–200. doi: 10.1158/1055-9965.EPI-15-1087 27197293

[77] Wada A Yamashita H Togashi A Ogawa S Muroi A Kido S. Combination of parenteral amino acid infusion and intermittent loading exercise ameliorates progression of postoperative sarcopenia in rat model. Nutrients. (2024) 16:1218. doi: 10.3390/nu16081218 38674908 PMC11054099

[78] Raitakari OT Porkka KVK Räsänen L Viikari JSA. Relations of life-style with lipids, blood pressure and insulin in adolescents and young adults. The Cardiovascular Risk in Young Finns Study. Atherosclerosis. (1994) 111:237–46. doi: 10.1016/0021-9150(94)90098-1 7718026

[79] Stanford KI Goodyear LJ. Exercise and type 2 diabetes: molecular mechanisms regulating glucose uptake in skeletal muscle. Adv Physiol Educ. (2014) 38:308–14. doi: 10.1152/advan.00080.2014 PMC4315445 25434013

[80] Kim J-S Wilson RL Taaffe DR Galvão DA Gray E Newton RU. Myokine Expression and Tumor-Suppressive Effect of Serum after 12 wk of Exercise in Prostate Cancer Patients on ADT. Med Sci Sports Exerc. (2022) 54:197–205. doi: 10.1249/MSS.0000000000002783 34559721 PMC8754092

[81] Febvey-Combes O Jobard E Rossary A Pialoux V Foucaut A-M Morelle M. Effects of an exercise and nutritional intervention on circulating biomarkers and metabolomic profiling during adjuvant treatment for localized breast cancer: results from the PASAPAS feasibility randomized controlled trial. Integr Cancer Ther. (2021) 20:153473542097766. doi: 10.1177/1534735420977666 PMC7934026 33655799

[82] Dieli-Conwright CM Courneya KS Demark-Wahnefried W Sami N Lee K Buchanan TA. Effects of aerobic and resistance exercise on metabolic syndrome, sarcopenic obesity, and circulating biomarkers in overweight or obese survivors of breast cancer: A randomized controlled trial. J Clin Oncol. (2018) 36:875–83. doi: 10.1200/JCO.2017.75.7526 PMC5858524 29356607

[83] Orange ST Leslie J Ross M Mann DA Wackerhage H. The exercise IL-6 enigma in cancer. Trends Endocrinol Metab TEM. (2023) 34:749–63. doi: 10.1016/j.tem.2023.08.001 37633799

[84] Zimmer P Bloch W Schenk A Zopf E Hildebrandt U Streckmann F. Exercise-induced natural killer cell activation is driven by epigenetic modifications. Int J Sports Med. (2015) 36:510–5. doi: 10.1055/s-0034-1398531 25714571

[85] Wennerberg E Lhuillier C Rybstein MD Dannenberg K Rudqvist N-P Koelwyn GJ. Exercise reduces immune suppression and breast cancer progression in a preclinical model. Oncotarget. (2020) 11:452–61. doi: 10.18632/oncotarget.27464 PMC6996907 32064049

[86] Abdalla DR Aleixo AAR Murta EFC Michelin MA. Innate immune response adaptation in mice subjected to administration of DMBA and physical activity. Oncol Lett. (2014) 7:886–90. doi: 10.3892/ol.2013.1774 PMC3919953 24520305

[87] Xiao Y Yu D. Tumor microenvironment as a therapeutic target in cancer. Pharmacol Ther. (2021) 221:107753. doi: 10.1016/j.pharmthera.2020.107753 33259885 PMC8084948

[88] Almeida PWM Gomes-Filho A Ferreira AJ Rodrigues CEM Dias-Peixoto MF Russo RC. Swim training suppresses tumor growth in mice. J Appl Physiol. (2009) 107:261–5. doi: 10.1152/japplphysiol.00249.2009 19478194

[89] Zielinski MR Muenchow M Wallig MA Horn PL Woods JA. Exercise delays allogeneic tumor growth and reduces intratumoral inflammation and vascularization. J Appl Physiol. (2004) 96:2249–56. doi: 10.1152/japplphysiol.01210.2003 PMC3645346 15020578

[90] Xiao Y Cong M Li J He D Wu Q Tian P. Cathepsin C promotes breast cancer lung metastasis by modulating neutrophil infiltration and neutrophil extracellular trap formation. Cancer Cell. (2021) 39:423–437.e7. doi: 10.1016/j.ccell.2020.12.012 33450198

[91] Gomes-Santos IL Amoozgar Z Kumar AS Ho WW Roh K Talele NP. Exercise training improves tumor control by increasing CD8+ T-cell infiltration via CXCR3 signaling and sensitizes breast cancer to immune checkpoint blockade. Cancer Immunol Res. (2021) 9:765–78. doi: 10.1158/2326-6066.CIR-20-0499 PMC8295193 33839688

[92] Rundqvist H Veliça P Barbieri L Gameiro PA Bargiela D Gojkovic M. Cytotoxic T-cells mediate exercise-induced reductions in tumor growth. eLife. (2020) 9:e59996. doi: 10.7554/eLife.59996 33095157 PMC7584454

[93] Kurz E Hirsch CA Dalton T Shadaloey SA Khodadadi-Jamayran A Miller G. Exercise-induced engagement of the IL-15/IL-15Rα axis promotes anti-tumor immunity in pancreatic cancer. Cancer Cell. (2022) 40:720–737.e5. doi: 10.1016/j.ccell.2022.05.006 35660135 PMC9280705

[94] Runowicz CD Leach CR Henry NL Henry KS Mackey HT Cowens-Alvarado RL. American cancer society/american society of clinical oncology breast cancer survivorship care guideline. CA Cancer J Clin. (2016) 66:43–73. doi: 10.3322/caac.21319 26641959

[95] Kim J-S Taaffe DR Galvão DA Hart NH Gray E Ryan CJ. Exercise in advanced prostate cancer elevates myokine levels and suppresses *in-vitro* cell growth. Prostate Cancer Prostatic Dis. (2022) 25:86–92. doi: 10.1038/s41391-022-00504-x 35152272 PMC8853098

[96] Thompson HJ Jiang W Zhu Z. Candidate mechanisms accounting for effects of physical activity on breast carcinogenesis. IUBMB Life. (2009) 61:895–901. doi: 10.1002/iub.233 19588523 PMC4346290

[97] Zhu Z Jiang W Sells JL Neil ES McGinley JN Thompson HJ. Effect of nonmotorized wheel running on mammary carcinogenesis: circulating biomarkers, cellular processes, and molecular mechanisms in rats. Cancer Epidemiol Biomarkers Prev. (2008) 17:1920–9. doi: 10.1158/1055-9965.EPI-08-0175 PMC2667869 18708381

[98] Xia Y Xu F Xiong M Yang H Lin W Xie Y. Repurposing of antipsychotic trifluoperazine for treating brain metastasis, lung metastasis and bone metastasis of melanoma by disrupting autophagy flux. Pharmacol Res. (2021) 163:105295. doi: 10.1016/j.phrs.2020.105295 33176207

[99] Schwappacher R Dieterich W Reljic D Pilarsky C Mukhopadhyay D Chang DK. Muscle-derived cytokines reduce growth, viability and migratory activity of pancreatic cancer cells. Cancers. (2021) 13:3820. doi: 10.3390/cancers13153820 34359731 PMC8345221

[100] Dethlefsen C Hansen LS Lillelund C Andersen C Gehl J Christensen JF. Exercise-induced catecholamines activate the hippo tumor suppressor pathway to reduce risks of breast cancer development. Cancer Res. (2017) 77:4894–904. doi: 10.1158/0008-5472.CAN-16-3125 28887324

[101] Zhang Q-B Zhang B-H Zhang K-Z Meng X-T Jia Q-A Zhang Q-B. Moderate swimming suppressed the growth and metastasis of the transplanted liver cancer in mice model: with reference to nervous system. Oncogene. (2016) 35:4122–31. doi: 10.1038/onc.2015.484 26686088

[102] Luo Z Mei J Wang X Wang R He Z Geffen Y. Voluntary exercise sensitizes cancer immunotherapy via the collagen inhibition-orchestrated inflammatory tumor immune microenvironment. Cell Rep. (2024) 43(9):114697. doi: 10.1016/j.celrep.2024.114697 39217611

[103] Luo Z Zhu J Xu R Wan R He Y Chen Y. Exercise-downregulated CD300E acted as a negative prognostic implication and tumor-promoted role in pan-cancer. Front Immunol. (2024) 15:1437068. doi: 10.3389/fimmu.2024.1437068 PMC11321962 39144140

[104] Luo Z Zhu J Fang Z Xu R Wan R He Y. Exercise-augmented THSD7B exhibited a positive prognostic implication and tumor-suppressed functionality in pan-cancer. Front Immunol. (2024) 15:1440226. doi: 10.3389/fimmu.2024.1440226 PMC11330788 39161765

[105] Luo Z Zhang T Chen S. Exercise Prescription: Pioneering the "Third Pole" for Clinical Health Management. Research (Wash D C). (2023) 6:0284. doi: 10.34133/research.0284 PMC10684289 38034085

[106] Luo Z Wan T Liu S Feng X Peng Z Wang Q. Mechanisms of exercise in the treatment of lung cancer - a mini-review. Front Immunol. (2023) 14:1244764. doi: 10.3389/fimmu.2023.1244764 PMC10483406 37691942

[107] Luo ZW Sun YY Xia W Xu JY Xie DJ Jiao CM. Physical exercise reverses immuno-cold tumor microenvironment via inhibiting SQLE in non-small cell lung cancer. Mil Med Res. (2023) 10(1):39. doi: 10.1186/s40779-023-00474-8 PMC10436398 37592367

